# Interaction between Apo A-II -265T>C polymorphism and dietary total antioxidant capacity on some anthropometric indices and serum lipid profile in patients with type 2 diabetes mellitus

**DOI:** 10.1017/jns.2020.61

**Published:** 2021-02-09

**Authors:** Banafsheh Jafari Azad, Mehdi Yaseri, Elnaz Daneshzad, Fariba Koohdani

**Affiliations:** 1 Department of Cellular and Molecular Nutrition, School of Nutritional Sciences and Dietetics, Tehran University of Medical Sciences, Tehran, Iran; 2 Department of Epidemiology and Biostatistics, School of Public Health, Tehran University of Medical Sciences, Tehran, Iran; 3 Department of Community Nutrition, School of Nutritional Sciences and Dietetics, Tehran University of Medical Sciences, Tehran, Iran; 4 Diabetes Research Center, Endocrinology and Metabolism Clinical Sciences Institute, Tehran University of Medical Sciences, Tehran, Iran

**Keywords:** Apolipoprotein A2, Dietary total antioxidant capacity, Interaction, Polymorphism, Antioxidant: Diabetes

## Abstract

The present study aimed to investigate the interaction of Apo A-II polymorphism and dietary total antioxidant capacity (DTAC) with lipid profile and anthropometric markers in patients with type 2 diabetes (T2DM) that are at risk for atherosclerosis. This cross-sectional study was conducted on 778 patients with T2DM (35–65 years). Dietary intakes were assessed by a 147-item food frequency questionnaire. DTAC was computed using international databases. Participants were categorised into two groups based on rs5082 genotypes. The gene–diet interaction was analysed by an ANCOVA multivariate interaction model. Total cholesterol, TC; triacylglycerol, TG; high- and low-density lipoprotein, HDL and LDL; TC–HDL ratio; waist circumference, WC and body mass index, BMI were obtained according to standard protocols. Overall, the frequency of CC homozygous was 12⋅1 % among study participants. We found that a significant interaction between rs5082 variants and DTAC on mean WC (*P*_TEAC_ = 0⋅044), TC concentration (*P*_FRAP_ = 0⋅049 and *P*_TEAC_ = 0⋅031) and TC/HDL (*P*_FRAP_ = 0⋅031 and *P*_TRAP_ = 0⋅040). Among patients whose DTAC was higher than the median intake, the mean of weight, WC and TC/HDL were significantly higher only in individuals with CC genotype. Also, the high DTAC was associated with a lower TC concentration only in T-allele carriers (*P*_FRAP_ = 0⋅042). We found that adherence to a diet with high total antioxidant capacity can improve the complications of diabetes and atherosclerosis in the T carrier genotype more effectively than the CC genotype. These results could indicate the anti-atherogenic properties of Apo A-II. However, further studies are needed to shed light on this issue.

## Introduction

Dyslipidemia and obesity have been recognised as a major health problem associated with diabetes mellitus (DM) and its complications^(1)^. Dyslipidemia is a condition identified by abnormal lipids profile (such as elevated blood concentrations of the low-density lipoprotein-cholesterol, LDL-c; triacylglycerol, TG and decreased high-density lipoprotein-cholesterol, HDL-c) in the blood^(2)^. In this regard, some studies indicated that high-blood TG level is considered as a risk factor for cardiovascular disease (CVD), particularly in the patient with diabetes^(3,4)^. It has been reported that the prevalence of diabetes in the Middle East will increase significantly and estimated that the prevalence of DM in Iran, as the second rank annual growth rate of diabetes in the region, will reach 9⋅2 million by 2030^(5,6)^. Several studies have shown that dyslipidemia and obesity are commonly observed in the Asian population and also is a major cause of CVD^(7–10)^.

Genetic differences play a significant role in the development of CVD^(11)^. The apolipoprotein loci (such as apolipoprotein A2, Apo A-II) are perhaps one of the best-studied genetic variables because of their relevance to CVD concerning dietary habits^(11)^. Apo A-II appeared to be a positional and biological candidate gene for type 2 DM (T2DM) at the chromosome 1q21–q24 susceptibility locus^(12)^. The amount of cardiometabolic risk factors varies between different variants of this polymorphism in the T2DM patients^(13–15)^. Some studies reported that Apo A-II -265T>C polymorphism is one of the single nucleotide polymorphisms (SNPs) that have been associated with anthropometric indices and plasma lipid concentration. A T-to-C substitution in the promoter region of this SNP resulted in decreased expression of Apo A-II in the liver in the CC genotype (as a minor allele) and subsequently decreased the plasma level of Apo A-II in this genotype^(16)^. Apo A-II is a constituent apolipoprotein of certain HDL particles and is a predictor of risk for CVD^(17)^. According to some studies, Apo A-II plays an important role in the regulation of appetite, cholesterol efflux, HDL remodelling, and metabolism of lipid and glucose^(18,19)^. And also it can influence the function of lipid transfer proteins and lipases^(13,19,20)^. Apo A-II concentrations have been reported to be associated with lipid profile concentration such as total cholesterol, LDL-c, HDL-c, TG^(12,21,22)^ and plasma clearance of large very-low-density lipoprotein (VLDL) particles^(16)^. Also, recent findings indicate that this apolipoprotein is associated with visceral obesity^(16,18,23)^ and body weight both in animals^(24,25)^ and human studies^(26,27)^.

Apo A-II -265T/C polymorphism affect lipid profile and fat metabolism^(16)^. In this regard, human studies indicated a higher lipid profile in the CC genotype^(12,21)^. Also, homozygous individuals for the -265C allele have been associated with increased obesity, visceral adipose tissue and body mass index (BMI) in several different populations^(11,18,28,29)^. This SNP has been related to lower waist circumference (WC) in healthy 50-year-old men^(16)^ and with higher waist-to-hip ratios in the T2DM individuals^(12)^. However, there are some studies regard to anthropometric measures with controversial results^(16,23,30)^. These inconsistent results may be attributed to dietary interactions that may have contributed to masking the influence of this SNP on lipid profile and anthropometric indices.

On the other hand, appropriate nutritional behaviour is a well-known environmental factor that can decrease the progression of diabetes and its complications like CVD. Researchers have shown that phytochemicals, as an antioxidant compound in food, decrease the risk of chronic disease through protect cells from oxidative damage^(7)^. Since many different antioxidant compounds coexist in whole foods, assessment of single antioxidants does not state the total antioxidant ability of the entire diet^(31)^. Dietary total antioxidant capacity (DTAC) is a common index for evaluation of total antioxidant content by considering the synergistic/interaction effects that may exist between the antioxidants in mixed food and is a useful indicator in epidemiological studies^(10,32,33)^. Some observational studies reported an inverse association between high DTAC and cardiometabolic risk factors in the different populations^(34–36)^. Different assays are available to estimate the DTAC^(37)^. It has been suggested that various methods should be used to provide clear and comprehensive information^(32)^.

Given that the APO A-II genotypes frequency in Iranian diabetic patients was reported to be 12 %^(13)^, the present study aimed to assess the interactions between this polymorphism and DTAC on anthropometric indices and lipid profile in the Iranian diabetic population to find an effective strategy to control, prevent or delay the progression of diabetes complications based on their allelic combination.

## Methods and materials

### Participants and study design

The sample size was defined according to the type I error of *α* = 0⋅05 and type II error of *β* = 80 %. In this cross-sectional study, 778 patients with T2DM (aged 35–65 years, both genders) were recruited. Participants were selected based on inclusion criteria randomly from the diabetes centres in Tehran. The Apo A-II -265T>C polymorphism, analysed by a real-time polymerase chain reaction, was previously genotyped for all patients by our research team (the frequency of the CC genotype was 12⋅9 %)^(14,38)^. Based on the previous studies in this area, genotype TT and TC was considered as T-allele carriers (TT + TC) and have been compared with CC^(14,15,28,39)^. According to this classification, we divided the patients into two groups of T-allele carriers (*n* 684) and C-allele homozygotes (*n* 94). Exclusion criteria including being a migrant, pregnant, lactation, on insulin therapy, and patients with clinical diseases such as coagulation disorders, cancers, stroke, malignant disease and patients with unexplained total energy intake (<800 or >4200 kcal/d)^(40)^. Demographic data and lifestyle variables such as age (year), gender, job status, smoking status (yes/no) and medical history (duration of diabetes and its complications, use of supplements or medications either lipid-lowering or glucose-lowering) were collected through pre-tested questionnaires. Subjects who smoked (daily or occasionally and passive smoker) were considered current smokers, while non-smoker included those who had never smoked. Also, information about the amount of daily physical activity was estimated in terms of metabolic equivalent × hours per day (MET h/d) using a physical activity questionnaire that its validity and reliability were confirmed in previous studies in Iran^(41)^. All T2DM patients provided informed written consent before participating in the study. The study protocol has been approved by the ethics committee of Tehran University of Medical Sciences (TUMS), with the following identification: 97-03-161-41169.

### Biochemical parameters and anthropometric measurements

Common anthropometric measures of patients (including height, weight, WC and BMI) were measured by a trained dietitian based on standard protocols. Height was recorded by using a non-elastic tape meter with an accuracy of 0⋅5 cm, while the patients were in standing posture, barefoot and shoulders in a relaxed position against the wall. Participants’ weight was measured in the fasting state, with no shoes and with minimal clothing by using a digital scale (Seca725 GmbH & Co., Hamburg, Germany) with an accuracy of 0⋅1 kg. WC was registered to the nearest 0⋅5 cm at the narrowest point between the last rib and the iliac crest. BMI was also calculated using the ‘weight (kg)/height^2^ (m)’ equation. Blood samples were taken in the morning, after 12-h fasting to measure serum lipids level (cholesterol, LDL, HDL and TG) by an enzymatic method (using kits, Pars Azmun Co., Iran). Cholesterol-to-HDL ratio and LDL-to-HDL ratio were also computed as the atherosclerotic index.

### Dietary assessment and definition of DTAC

The habitual diet during the preceding year was ascertained using a validated semi-quantitative food frequency questionnaire (semi-FFQ) with 147 food items^(42)^. Trained staff asked participants to designate their intake frequency for each food item consumed on a daily, weekly, monthly, or annually basis. Then, the portion size of consumed foods was expressed in grams using household measures. Due to the unavailability of the database to calculate the amount of antioxidants in Iranian food, we used the most common assays^(37)^ and based on the previously published databases that contained most of the foods that are consumed by the Iranian population, including FRAP based on Norwegian antioxidant table that includes more than 3000 foods^(43)^; ORAC based on the United State Department of Agriculture (USDA) databases^(44)^; and TEAC and TRAP that were based on Italian food database^(45)^. FRAP values are expressed as millimoles Fe^2+^/100 g of food. Two other methods (TEAC and TRAP) are expressed as millimole of Trolox equivalent (mmol TE)/kg of food and micromole of Trolox equivalent (μmol TE)/100 g of food for ORAC methods, respectively^(46)^. If DTAC data were not matched for any food item, the value of the nearest comparable food was assigned. Also, we used the mean value of similar foods (e.g. several types of bread)^(47)^. Antioxidants from supplements were not included in the calculation of DTAC^(32)^. The daily intake (gram) of each selected food item was multiplied by their related antioxidant content (FRAP, TRAP, ORAC and ORAC) values per food portion from the database and then summed up to estimate DTAC for each participant^(32)^. Finally, DTAC is categorised based on the median intake (low, ≤median and high, >median).

### Statistical methods

Subjects were divided into two Apo A-II genotype groups: CC- or T-allele carriers (TT+TC). Also, according to the median DTAC, the participants were dichotomised into ‘high’ and ‘low’ categories (≤, <median): FRAP (≤15⋅94, 15⋅95 > mmol Fe^2+^/d); TRAP (≤8⋅25, 8⋅26 > mmol TE/d); TEAC (≤7⋅46, 7⋅47 > mmol TE/d) and ORAC (≤27 296⋅21, 27 296⋅22 > μmol TE/d). Normality distribution was checked using the Kolmogorov–Smirnov's test. Logarithmic transformations were applied to variables with non-normal distributions. To compare qualitative (difference in the percentage) and quantitative (difference in the means) variables with normal distributions between the two groups (CC and TC + TT groups/low and high DTAC), the *χ*^2^ and Independent Student's *t* test were utilised, respectively. Analysis of covariance (ANCOVA) test was applied to compare the mean dependents variables with adjusting for confounding variables. ANCOVA multivariate interaction models using the general linear model was tested to find the interaction between Apo A-II polymorphism (rs5082) and DTAC on anthropometric indices and lipid profile. Additional adjustments were done for confounder variables including age (years), gender (male/female), smoking (yes/no), supplement use (yes/no), physical activity (continuous), lipid-lowering medication (yes/no), fibre (continuous) and total energy (continuous) intake. The data were presented as mean ± sd for normally distributed continuous variables and data that were not normally distributed were expressed as median (25th and 75th percentile). Categorical variables (qualitative) were reported as a percentage. Also, interactions were presented as a graph to help their illustration. *P*-value of less than 0⋅05 was considered significant. Statistical Package for the Social Sciences (SPSS Inc., Chicago, IL, USA, version 21) was employed for all steps of the statistical analysis. Variance inflation factor (VIF > 2), as a determinant of the presence of collinearity, was not used in the models.

## Results

### Study population characteristics

We studied the evaluated 778 diabetic patients in the present study. Table 1 shows the general characteristics based on each participant's genotype. According to our findings, genotype frequency among study participants was as follows: CC (12⋅1 %) and TC + TT (87⋅9 %). Gender and age distribution of T2DM population was (male = 39⋅4 %; female = 60⋅6 %, with a mean age of 55⋅0 ± 6) in CC genotype and (male = 39⋅2 %; female = 60⋅8 %, with a mean age of 54⋅0 ± 7) in T-allele carriers. Mean age was significantly higher in the CC genotype compared to T-allele carriers (TT + TC) (*P* = 0⋅023), and also, smoking is significantly more common in T-allele carriers (*P* = 0⋅027). Therefore, the null hypothesis was rejected. No significant difference was identified in the physical activity, dietary intake, glucose-lowering and lipid-lowering medication intake between the two genotype groups. Moreover, the percentage of FFQ items with antioxidant capacity values assigned in the database the compliance of our FFQ questionnaire items with international databases varied among the analysed methods (FRAP = 80 %; TRAP = 41 %; TEAC = 46 %; ORAC = 49 %). In other studies, the percentage ranged from 44⋅8^(48)^ to 100⋅0 %^(49)^.

**Table 1.** Characteristics of patients with type 2 diabetes mellitus^a^

Variables	Apo A-II genotype	
	CC	TT + TC
Number of participants, *n* (%)	94 (12⋅1)	684 (87⋅9)
Age (year)	55 ± 6	54 ± 7
Male/ Female, *n* (%)	37/57 (39⋅4/60⋅6)	268/415 (39⋅2/60⋅8)
Smoking, Yes/No, *n* (%)	10/84 (10⋅6/89⋅4)	138/545 (20⋅2/79⋅8)
Physical activity (MET h/d)	36⋅96 ± 1⋅12	37⋅33 ± 1⋅15
Medical history		
Diabetes duration (≤5 years), *n* (%)	35 (37⋅2)	305 (44⋅7)
Diabetes duration (>5 years), *n* (%)	59 (62⋅8)	378 (55⋅3)
History of CVD, Yes/No, *n* (%)	37/57 (39⋅4/60⋅6)	256/427 (37⋅5/62⋅5)
Supplement use, Yes/No, *n* (%)^b^	47/47 (50/50)	333/345 (49⋅1/50⋅9)
Lipid-lowering medication, Yes/No, *n* (%)^c^	56/38 (59⋅6/40⋅4)	390/292 (57⋅2/42⋅8)
Glucose-lowering medication, Yes/No, *n* (%)^d^	88/6 (93⋅6/6⋅4)	635/48 (93/7)
Food intake		
Energy (kcal/d)	2416⋅31 ± 1⋅43	2465⋅13 ± 1⋅37
Carbohydrate (g/d)	323⋅75 ± 1⋅46	320⋅53 ± 1⋅40
Protein (g/d)	83⋅93 ± 1⋅46	84⋅77 ± 1⋅39
Fat (g/d)	92⋅75 ± 1⋅53	94⋅63 ± 1⋅53
Fibre (g/d)	40⋅04 ± 1⋅53	38⋅09 ± 1⋅55
Dietary total antioxidant capacity		
FRAP (mmol Fe^2^^+^/100 g)	15⋅48 ± 3⋅27	15⋅48 ± 3⋅18
TRAP (mmol TE/kg)	8⋅00 ± 1⋅35	8⋅67 ± 1⋅58
TEAC (mmol TE/kg)	7⋅38 ± 1⋅49	7⋅84 ± 1⋅52
ORAC (μmol TE/100 g)	28 853⋅88 ± 1⋅50	27 722⋅57 ± 1⋅44

Abbreviations: CVD, cardiovascular disease; MET, metabolic equivalents; FRAP, ferric reducing ability of plasma; TRAP, total reactive antioxidant potential; TEAC, Trolox equivalent antioxidant capacity; ORAC, oxygen radical absorbance capacity.

a All data are mean ± sd unless indicated.

b Supplements of vitamins and minerals.

c Statins, gemfibrozil and nicotinamide.

d Metformin, glibenclamide and thiazolidionedione.

### Interaction between the Apo A-II -265T/C SNP and the DTAC in determining anthropometric variables

The interaction between the Apo A-II -265T>C polymorphism and the DTAC (FRAP, TRAP, TEAC and ORAC) on anthropometric indices (WC and BMI) is illustrated in Fig. 1(A) and 1(B). No significant difference was observed in the mean BMI of patients with CC genotype and patients with T-allele carrier genotype (TT + TC) in different categories of DTAC. Although unlike patients with CC genotype, in patients with T allele, the mean BMI in the group with higher DTAC was numerically lower than in patients with lower DTAC, but this difference was not statistically significant. Also, no significant interaction was seen between the gene polymorphism and the DTAC on the BMI of patients with type 2 diabetes (Fig. 1(A)). The results of the analysis showed that in the CC genotype, the mean of WC in subjects with higher than the median intake of DTAC was higher than subjects with lower than median intake. This direct association between higher DTAC and mean WC in the CC genotypic group was statistically significant only in the TEAC method (*P* = 0⋅01). Furthermore, a gene–diet interaction was detected between DTAC by TEAC and Apo A-II rs5082 on WC (*P*_Interaction_ = 0⋅044). The statistical adjustment did not change the statistical significance of these gene–diet interactions (*P*_1_ = 0⋅045, *P*_2_ = 0⋅037) (Fig. 1(B)).

**Fig. 1A.**
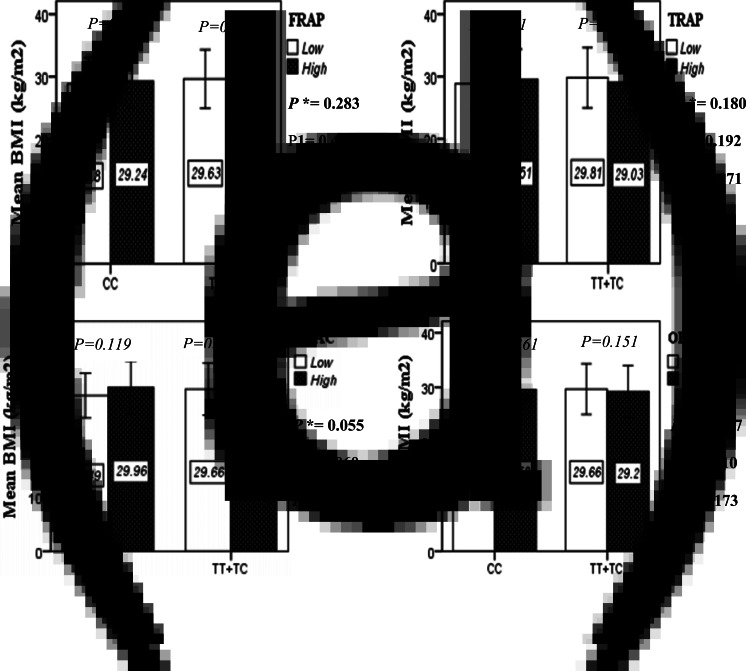
Interaction between the APOA2 polymorphism and the dietary TAC: FRAP (a), TRAP (b), TEAC (c), and ORAC (d) intake with regard to BMI. According to the median dietary TAC the participants were dichotomized into “high” and “low” categories (≤, > of median), FRAP (≤15.94, 15.95 > mmol Fe^2+^/d); TRAP (≤8.25, 8.26 > mmol TE/d); TEAC (≤7.46, 7.47> mmol TE/d); ORAC (≤27296.21, 27296.22> μmol TE/d). P*; crude, P1; model 1, and P2; model 2. P*-values for the interaction terms between dietary TAC intake (as dichotomous) and the APOA2 polymorphism were obtained with General Linear Model (Two-Way ANOVA). The P1 value of the interaction (Model 1) is adjusted for supplement use (as categorical), smoking (as categorical), and total energy intake (as continuous). In model 2, in addition to the variables of model 1, it was also adjusted based on the variables of age (as continuous) and sex (as categorical) using the ANCOVA test. In the stratified analysis by APOA2 genotypes, P-values for mean comparisons of BMI between two categories of antioxidant intake were estimated by Independent Samples t-test. Bars indicate mean ± SD.

**Fig. 1B.**
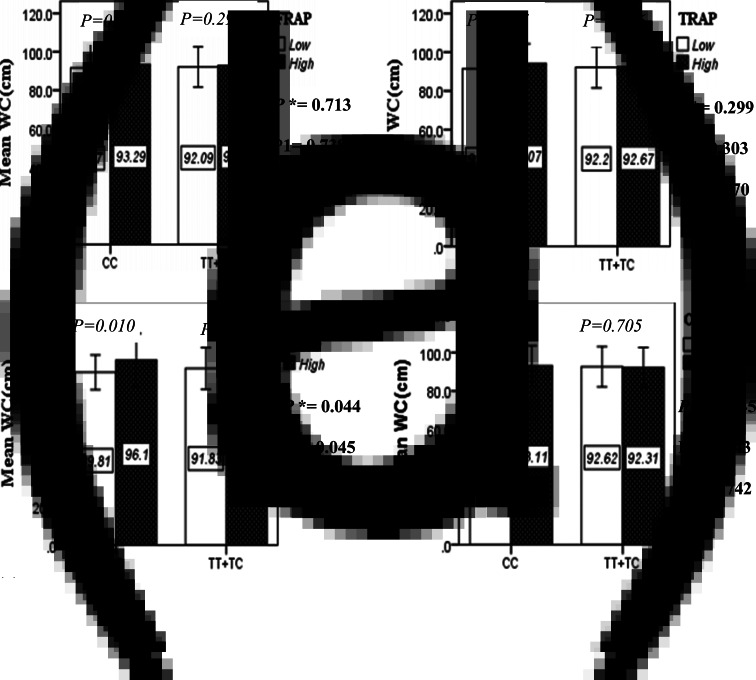
The interaction between APOA2 -265 T>C polymorphism and the dietary TAC: FRAP (a), TRAP (b), TEAC (c), and ORAC (d) intake on WC. According to the median dietary TAC the participants were dichotomized into “high” and “low” categories (≤, > of median), FRAP (≤15.94, 15.95 > mmol Fe^2+^/d); TRAP (≤8.25, 8.26 > mmol TE/d); TEAC (≤7.46, 7.47> mmol TE/d); ORAC (≤27296.21, 27296.22> μmol TE/d). P*; crude, P1; model 1, and P2; model 2. P*-values for the interaction terms between dietary TAC intake (as dichotomous) and the APOA2 polymorphism in each population were obtained in the General Linear Model (Two-Way ANOVA). The P1 value of the interaction (Model 1) is adjusted for supplement use (as categorical), smoking (as categorical), and total energy intake (as continuous). In model 2, in addition to the variables of model 1, it was also adjusted based on the variables of age (as continuous) and sex (as categorical) using the ANCOVA test. Independent Samples t-test was used to compare the mean WC between low and high dietary TAC intake base on rs5082 genotypes. The bars indicate mean ± SD.

### Interactions between the Apo A-II -265T/C SNP and the DTAC in determining lipid profile

The interaction between the Apo A-II -265T>C polymorphism and the DTAC (FRAP, TRAP, TEAC and ORAC) on lipid profile (TC, TG, LDL, HDL and TC/HDL) is illustrated in Fig. 2(A)–(E). In T-allele carriers, higher DTAC (FRAP method) was significantly related to decreased serum levels of TC (*P* = 0⋅042) (Fig. 2A-a). However, in patients with CC genotype, no significant difference was observed in the serum levels of TC between the two groups of low and high intake of DTAC. Only in the TEAC method, the interaction between the Apo A-II polymorphism and the DTAC on the serum level of TC was statistically significant in both crude and adjusted models (*P*_Interaction_ = 0⋅031, *P*_1_ = 0⋅032, *P*_2_ = 0⋅034) (Fig. 2(A-c)). Moreover, the interaction of gene and antioxidant intake in the FRAP method was significant in crude (*P* = 0⋅049) and also adjusted model 1 (*P*_1_ = 0⋅045), but in model 2, this significant interaction was lost (*P*_2_ = 0⋅069) (Fig. 2(A-a)). However, this interaction was not significant in the ORAC and TRAP methods in either the crude or adjusted models (Fig. 2(A)). There was no significant interaction between rs5082 genotypes and DTAC on the serum level of TG, LDL and HDL in the crude and adjustment models (Fig. 2(B)–2(D)). In patients with the CC genotype, higher DTAC was significantly related to the increased TC–HDL ratio and this association was significant only in the TRAP method (*P* = 0⋅032) (Fig. 2(E-b)). However, in T-allele carriers, no significant difference was observed in the TC–HDL ratio between the two groups of low and high intake of DTAC. The interaction of gene and antioxidant intake on the TC–HDL ratio in FRAP and TRAP methods were significant in crude (*P*_FRAP_ = 0⋅031, *P*_TRAP_ = 0⋅040) and also adjusted model 1 (*P*_1FRAP_ = 0⋅034, *P*_1TRAP_ = 0⋅047). The significant interaction persisted on even after adjusting potential confounders (*P*_2FRAP_ = 0⋅045) (Fig. 2(E)).

**Fig. 2A.**
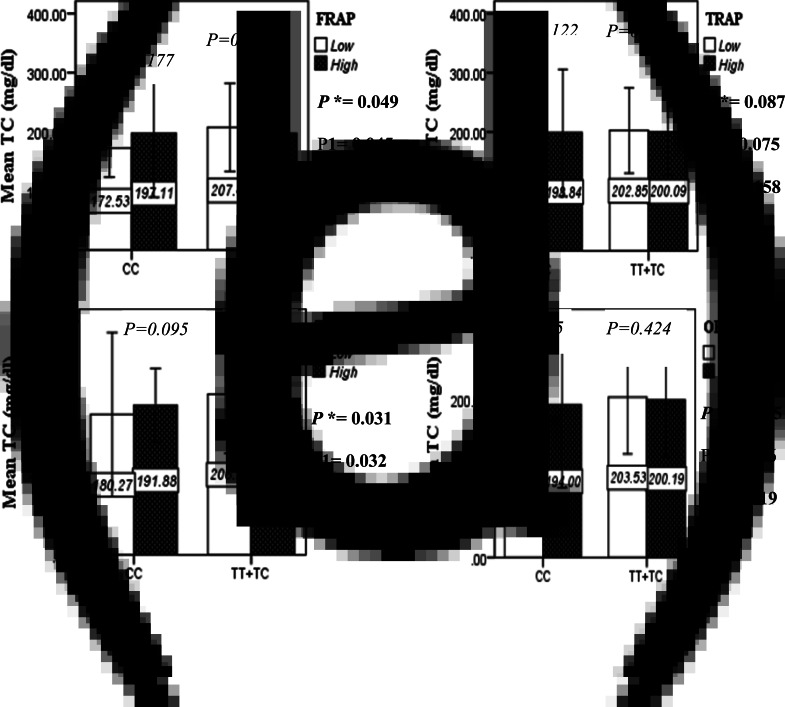
Interaction between APOA2 −265T>C polymorphism and the DTAC: FRAP (a), TRAP (b), TEAC (c), and ORAC (d) intake on serum total cholesterol level. According to the median dietary TAC the participants were dichotomized into “high” and “low” categories (≤, > of median), FRAP (≤15.94, 15.95 > mmol Fe^2+^/d); TRAP (≤8.25, 8.26 > mmol TE/d); TEAC (≤7.46, 7.47> mmol TE/d); ORAC (≤27296.21, 27296.22> μmol TE/d). P*; crude, P1; model 1, and P2; model 2. P* interactions are obtained with the General Linear Model (Two-Way ANOVA). The P1 value of the interaction (Model 1) is adjusted for sex (as categorical), supplement use (as categorical), smoking (as categorical), fiber, and total energy intake (as continuous). In model 2, in addition to the variables of model 1, it was also adjusted based on the variables of age (as continuous), lipid-lowering medicine (as categorical) and BMI (as continuous) using the ANCOVA test. Independent Samples t-test was used to compare the serum total cholesterol level in the two categories of antioxidant intake. The bars indicate mean (SD).

**Fig. 2B.**
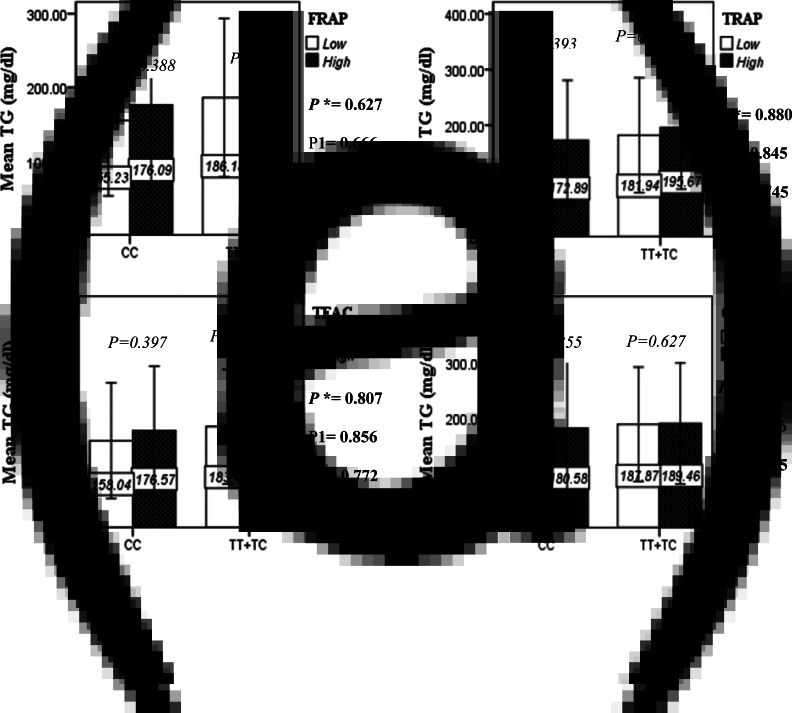
Interaction between the APOA2 polymorphism and the DTAC: FRAP (a), TRAP (b), TEAC (c), and ORAC (d) intake with regard to serum triglyceride level. According to the median dietary TAC the participants were dichotomized into “high” and “low” categories (≤, > of median), FRAP (≤15.94, 15.95 > mmol Fe^2+^/d); TRAP (≤8.25, 8.26 > mmol TE/d); TEAC (≤7.46, 7.47> mmol TE/d); ORAC (≤27296.21, 27296.22> μmolTE/d). P*; crude, P1; model 1, and P2; model 2. P*-values for the interaction terms between dietary TAC intake (as dichotomous) and the APOA2 polymorphism were obtained with General Linear Model (Two-Way ANOVA). The P1 value of the interaction (Model 1) is adjusted for sex(as categorical),supplement use (as categorical), and fiber, carbohydrate total energy intake (as continuous). In model 2, in addition to the variables of model 1, it was also adjusted based on the variables of age (as continuous) and smoking (as categorical), lipid-lowering medicine (as categorical) and BMI (as continuous) using the ANCOVA test. In the stratified analysis by APOA2 genotypes, P-values for mean comparisons of serum triglyceride levels between two categories of antioxidant intake were estimated by Independent Samples t-test. Bars indicate SD.

## Discussion

In this cross-sectional study, we observed a significant interaction between the Apo A-II polymorphism and the DTAC on mean WC (TEAC method), TC (FRAP and TEAC methods) and TC/HDL (FRAP and TRAP) in the diabetic patients. In the current survey, the increased mean of WC (significant only in the TEAC method), and TC/HDL (significant only in the TRAP method) were related to a higher median intake of DTAC in the CC genotype. Also, when stratified by rs5082 genotypes, the high DTAC was only associated with a lower TC concentration in T-allele carriers and this relationship is significant only in the FRAP method. Based on these findings, it seems that the presence of the T allele in diabetic patients with high DTAC is a protective factor against cardiometabolic risk factors. These results could indicate the anti-atherogenic properties of Apo A-II. Therefore, individuals with CC genotype are prone to cardiovascular disorders despite adherence to a diet rich in antioxidants.

**Fig. 2C.**
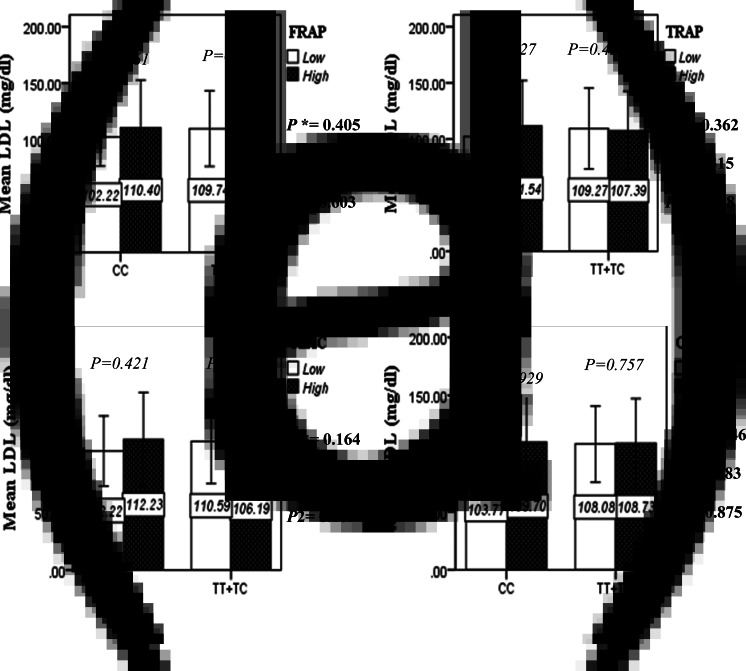
The interaction between APOA2 -265 T>C polymorphism and the DTAC: FRAP (a), TRAP (b), TEAC (c), and ORAC (d) intake on serum LDL level. According to the median dietary TAC the participants were dichotomized into “high” and “low” categories (≤, > of median), FRAP (≤15.94, 15.95 > mmol Fe^2+^/d); TRAP (≤8.25, 8.26 > mmol TE/d); TEAC (≤7.46, 7.47> mmol TE/d); ORAC (≤27296.21, 27296.22> μmol TE/d). P*; crude, P1; model 1, and P2; model 2. P*-values for the interaction terms between dietary TAC intake (as dichotomous) and the APOA2 polymorphism in each population were obtained in the General Linear Model (Two-Way ANOVA). The P1 value of the interaction (Model 1) is adjusted for sex (as categorical), supplement use (as categorical), PUFA, fiber, and total energy intake (as continuous). In model 2, in addition to the variables of model 1, it was also adjusted based on the variables of age (as continuous), BMI (as continuous), smoking (as categorical), and lipid-lowering medicine (as categorical) using the ANCOVA test. Independent Samples t-test was used to compare the mean serum LDL level between low and high DTAC intake base on rs5082 genotypes. The bars indicate mean (SD).

Although the precise mechanism by which rs5082 SNP interacts with DTAC is largely unknown, there are some lines of evidence supporting our findings. The prevailing view is that the function of HDL rather than its plasma concentration is more applicable for the evaluation of atherosclerotic risk. The functionality of HDL depends on their apolipoprotein composition^(50)^. Apo A-II is the second most abundant structural apolipoprotein related to HDL. This apolipoprotein is considered pro-atherogenic by some researchers and anti-atherogenic by others^(51,52)^. Few genetic variants have been detected in the Apo A-II gene on the 1q21–q23 chromosome^(53)^. The functional -265T/C SNP (rs. no. 5082) is located in the middle of element D of the Apo A-II gene promoter, which is linked to several different nuclear factors and probably interrupts the delicate balance of binding of these nuclear factors^(16,18)^. It has been reported that basal Apo A-II transcription and its concentration decreased in the CC homozygote in comparison with the T-allele carrier groups^(21)^. van't Hooft *et al*. have stated that linkage disequilibrium between the -265T/C SNP and another functional polymorphism in the human Apo A-II gene (such as MspI and BstNI) is unlikely. But, observed associations may be affected by functional polymorphisms in other genes^(16)^.

Some documents have implied that Apo A-II itself affects directly specific anti-atherogenic pathways^(54–56)^. One probable mechanism may be due to promoting cholesterol efflux through increase activity of lecithin cholesterol acyltransferase^(57,58)^. Also, amphipathic α-helices as structural domains of the Apo A-II are related to anti-atherogenic functions^(59)^. Some human studies indicated that inflammation and oxidative status are higher in diabetic patients with CC genotype than the carrier T allele^(15,60)^. In contrast, it has been reported that Apo A-II via inhibition of enzymatic activities lipoprotein and hepatic lipase leads to increased TG^(21)^. However, we founded that there is no significant association in serum TG in CC groups and T carrier groups, across two categories of DTAC. Also, some animal studies which have been conducted on transgenic mice indicated that overexpression of human Apo A-II *per se* results in a pro-atherogenic phenotype by means of an increase in cholesterol and/or triacylglycerol ApoB-containing lipoproteins^(61–64)^. However, it has been stated that mice are not the best animal model for examining human Apo A-II metabolism^(65,66)^.

**Fig. 2D.**
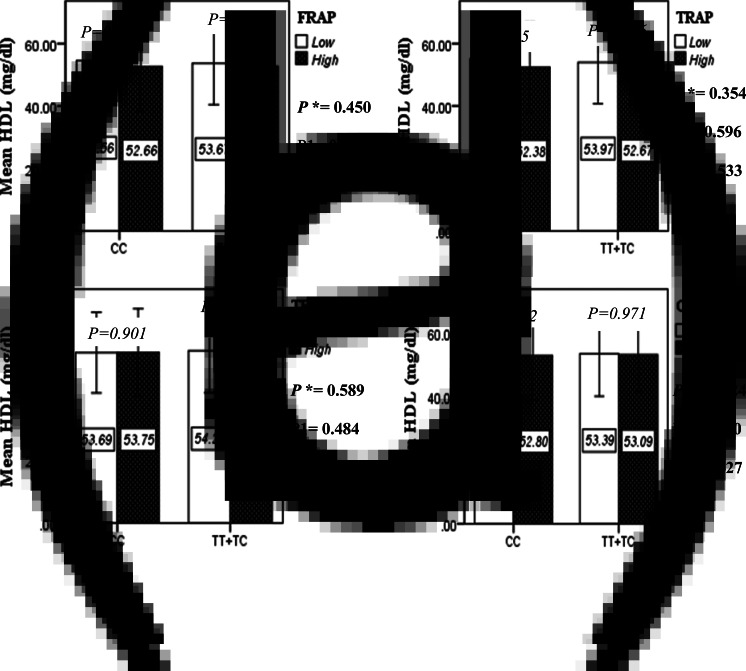
Interaction between the APOA2 polymorphism and the DTAC: FRAP (a), TRAP (b), TEAC (c), and ORAC (d) intake with regard to serum HDL level. According to the median dietary TAC the participants were dichotomized into “high” and “low” categories (≤, > of median), FRAP (≤15.94, 15.95 > mmol Fe^2+^/d); TRAP (≤8.25, 8.26 > mmol TE/d); TEAC (≤7.46, 7.47> mmol TE/d); ORAC (≤27296.21, 27296.22> μmol TE/d). P*; crude, P1; model 1, and P2; model 2. P*-values for the interaction terms between dietary TAC intake (as dichotomous) and the APOA2 polymorphism were obtained with General Linear Model (Two-Way ANOVA). The P1 value of the interaction (Model 1) is adjusted for age (as continuous) sex (as categorical), smoking (as categorical), and physical activity (as categorical). In model 2, in addition to the variables of model 1, it was also adjusted based on the variables of supplement use (as categorical), lipid-lowering medicine (as categorical), BMI (as continuous), and total energy intake (as continuous) using the ANCOVA test. In the stratified analysis by APOA2 genotypes, P-values for mean comparisons of serum HDL level between two categories of antioxidant intake were estimated by Independent Samples t-test. Bars indicate SD.

As mentioned before, oxidative stress plays an important role in the pathophysiology of diabetes and its complications such as micro- and macrovascular. In this regard, heat-shock proteins (HSPs) function in the *de novo* synthesis of proteins and repair damaged proteins which are induced by various stresses. Among the HSPs family, HSP60 is a highly conserved protein that confers protection against cardiac ischemia-reperfusion injury^(67)^. One animal study indicated that α-lipoic acid as a natural thiol antioxidant modifies HSP60 gene expression *in vivo* and protects rat tissues against oxidative stress^(68)^. HSP60 has been reported to be a high-affinity HDL-binding protein, particularly via binding of Apo A-II^(52)^. Some human researches indicated that homozygous patients for the -265C allele have higher obesity-related measures than did carriers of the T allele^(16,28,69)^. One probable mechanism may be due to the association of this apolipoprotein with satiety signals. Corella *et al*. were the first to report that Apo A-II has a role in the regulation of dietary intake and human appetite^(18)^. In this regard, we have previously reported in diabetic patients that the Apo A-II -265T/C SNP was associated with increased levels of serum ghrelin hormone^(14)^. However, we observed no significant interaction between DTAC and Apo A-II genotypes on BMI.

**Fig. 2E.**
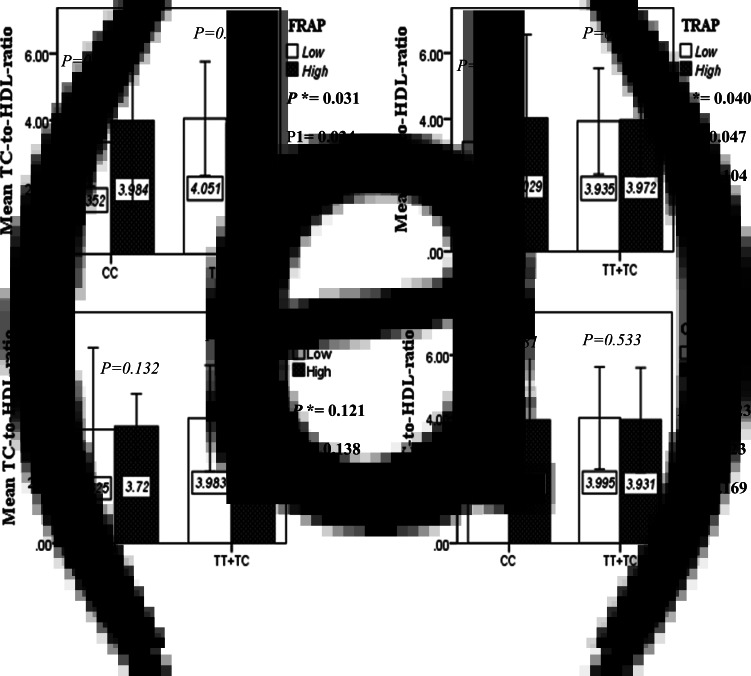
The interaction between APOA2 -265 T>C polymorphism and the DTAC: FRAP (a), TRAP (b), TEAC (c), and ORAC (d) intake on TC- HDL-ratio. According to the median dietary TAC the participants were dichotomized into “high” and “low” categories (≤, > of median), FRAP (≤15.94, 15.95 > mmol Fe^2+^/d); TRAP (≤8.25, 8.26 > mmol TE/d); TEAC (≤7.46, 7.47> mmol TE/d); ORAC (≤27296.21, 27296.22> μmol TE/d). P*; crude, P1; model 1, and P2; model 2. P*-values for the interaction terms between dietary TAC intake (as dichotomous) and the APOA2 polymorphism in each population were obtained in the General Linear Model (Two-Way ANOVA). The P1 value of the interaction (Model 1) is adjusted for age (as continuous) and smoking (as categorical), fiber, and total energy intake (as continuous). In model 2, in addition to the variables of model 1, it was also adjusted based on the variables of sex (as categorical), supplement use (as categorical), lipid-lowering medicine (as categorical), and BMI (as continuous) using the ANCOVA test. Independent Samples t-test was used to compare the mean TC/HDL between low and high DTAC intake base on rs5082 genotypes. The bars indicate mean (SD).

On the effect of diet on the human genome, some nutrigenomic studies pointed out the interactions between a Mediterranean diet as an antioxidant-rich dietary pattern and Apo A-II gene polymorphism^(69,70)^. In this research, we observed that a diet with high total antioxidant capacity can be more effective in the T carrier genotype than the CC genotype. Consistent with our results, Moradi *et al*. in an intervention study indicated that losing weight through calorie restriction for 6 weeks can ameliorate the conditions more efficiently in diabetic patients of the -265T allele carriers compared to the CC genotype^(71)^. The aforementioned study stated that T-allele carriers are more sensitive to lifestyle modification. Diet rich in antioxidants may change the unknown metabolic pathways which lead to altering susceptibility to CVD. Some epidemiological studies have shown the favourable effect of antioxidant-rich diets on components of metabolic syndrome^(36)^, CVD risk factors^(33)^ and obesity-related features^(72)^. The possible mechanism may be due to decreased inflammation and oxidative stress, reduce the absorption of fats and cholesterol from the intestine, increase the gene expression leptin, regulation of appetite, regulation of adipocyte metabolism and inhibition of nuclear factor-κB factor^(31,33,73)^. Moreover, it has been reported that lipid-soluble antioxidants such as polyphenols and carotenoids could inhibit cholesterol synthesis, increase faecal bile excretion, and have the ability to stimulate the expression of LDL receptors^(34)^. Moreover, the fibre in foods rich in antioxidants and also short-chain fatty acids resulting from the fermentation fibre in the intestine may be effective on moderate weight through inducing a sense of satiety and stimulate the peroxisome proliferator-activated receptor-a pathway (PPAR-a), respectively^(74,75)^. However, some studies observed no association between the highest quartile of DTAC and anthropometric indices^(76–78)^ or lipid profile^(34,78)^. The lack of association observed in some studies maybe is due to using only one assay to estimate DTAC, ignoring some potential confounding variables, and may also is related to did not capture the usual dietary intake (using a 24-h recall instead of an FFQ)^(32,79)^.

As mentioned earlier, a gold standard method for measuring the DTAC is not available yet. It has been suggested that various methods should be applied to provide clear and comprehensive information. In the present study, DTAC was estimated using four methods. Discrepancies between methods may be explained by the differences in the calculation methods and their chemical background. Also, these four databases include different numbers of foods such as vegetables, fruits, nuts and dried fruits^(80)^. Generally, antioxidants can neutralise radicals through two mechanisms, hydrogen atom transfer (HAT), which is based on the inhibition of peroxyl radical production, and single electron transfer (SET), which is based on the transfer of a single electron from an antioxidant molecule to an oxidant particle. FRAP and TEAC methods are based on the HAT reaction mechanisms. ORAC and TRAP methods are based on the SET reaction mechanisms^(81)^. In our study, the most significant interactions were detected in SET-based methods, particularly in the TEAC method. In this method, the antioxidant power in both hydrophilic and hydrophobic environments is calculated at the DTAC score. While lipophilic antioxidants are not calculated in TRAP and FRAP assays^(82)^.

Based on our study, adherence to a diet with high total antioxidant capacity may be considered as an effective strategy to reduce the risk of CVD in the T carrier group. Also, it seems that the antioxidant capacity of diet could not compensate for Apo A-II deficiency in diabetic patients with CC genotype. In other words, in the field of health services management, patients with CC genotype should be given more attention. Also, to prevent and control the progression of diabetes complications in this genotype group, other effective strategies and programmes are needed. In this regard, two studies have shown that higher intake of anti-inflammatory fatty acids, such as *n*-3 PUFA and MUFA could compensate for inflammatory effects caused by low plasma level of Apo A-II in CC genotype^(83,84)^. Studies have shown that dietary intake of W3 fatty acids, like antioxidants, inhibits inflammation, with the difference that, antioxidants exert this anti-inflammatory effect by inhibiting nuclear factor-κB and W3 fatty acid by inhibiting toll-like receptor^(85)^. It seems that a diet rich in antioxidants and anti-inflammatory fatty acids can be effective in reducing the risk of CVD in diabetic patients with T allele and CC genotype, respectively. The present study provides evidence for designing genotype-based dietary recommendations.

### Limitation

The present study is the first attempt to examine the association between Apo A-II polymorphism and DTAC, as measured by a variety of DTAC assays, in the Middle East. We also selected the different methods to estimate DTAC (FRAP, TRAP, TEAC and ORAC) to capture the multidimensionality of antioxidant potential. Moreover, other strengths of the study are the large number of participants.

Our study has some limitations. First, the cross-sectional nature prevented us from inferring causation but is useful in hypothesis generation. Second, due to the total antioxidant capacity that has not been measured for Iranian foods, DTAC was estimated based on international databases. Since the antioxidant content may vary according to geographic location, cultivation methods used, storage conditions, it is possible that this approach leads to an underestimation of TAC, attenuation, or invalidity of the assessed association. Third, when no data were available regarding cooked foods (typically have higher DTAC values), the value of raw foods was substituted. Fourth, it should be mentioned that the FFQ used for the assessment of participants’ dietary habits was not particularly validated for the estimation of diet's TAC. Fifth, DTAC did not consider antioxidant bioavailability or metabolism. Sixth, we lacked data on blood glucose, socioeconomic status, depression status, body composition, alcohol consumption, oxidative stress and inflammatory markers as a potential moderator of CVD risk and therefore could not control for these variables. Seventh, we did not consider the intake of dietary supplements in calculating DTAC. Also, lipid parameter measurements were collected in the fasted state, which may mask variations in Apo A-II under fed conditions. A major limitation associated with this investigation is that the increase in family-wise error rate across the reported statistical analysis was not controlled. Overall, we consider this research relatively preliminary and encourage replication.

## Conclusion

In summary, we found significant interactions between rs5082 variants and DTAC intake on mean WC, TC concentration and TC/HDL. Based on these results, the presence of the T allele in diabetic patients with high DTAC is a protective factor against cardiometabolic risk factors. These results could indicate the anti-atherogenic properties of Apo A-II. Future studies are needed to confirm these results.
